# Validation of Coevolving Residue Algorithms via Pipeline Sensitivity Analysis: ELSC and OMES and ZNMI, Oh My!

**DOI:** 10.1371/journal.pone.0010779

**Published:** 2010-06-01

**Authors:** Christopher A. Brown, Kevin S. Brown

**Affiliations:** 1 Department of Chemistry and Chemical Biology, Harvard University, Cambridge, Massachusetts, United States of America; 2 FAS Center for Systems Biology, Harvard University, Cambridge, Massachusetts, United States of America; 3 Department of Physics, University of California Santa Barbara, Santa Barbara, California, United States of America; 4 Institute for Collaborative Biotechnologies, University of California Santa Barbara, Santa Barbara, California, United States of America; University of Manchester, United Kingdom

## Abstract

Correlated amino acid substitution algorithms attempt to discover groups of residues that co-fluctuate due to either structural or functional constraints. Although these algorithms could inform both *ab initio* protein folding calculations and evolutionary studies, their utility for these purposes has been hindered by a lack of confidence in their predictions due to hard to control sources of error. To complicate matters further, naive users are confronted with a multitude of methods to choose from, in addition to the mechanics of assembling and pruning a dataset. We first introduce a new pair scoring method, called ZNMI (Z-scored-product Normalized Mutual Information), which drastically improves the performance of mutual information for co-fluctuating residue prediction. Second and more important, we recast the process of finding coevolving residues in proteins as a data-processing pipeline inspired by the medical imaging literature. We construct an ensemble of alignment partitions that can be used in a cross-validation scheme to assess the effects of choices made during the procedure on the resulting predictions. This pipeline sensitivity study gives a measure of reproducibility (how similar are the predictions given perturbations to the pipeline?) and accuracy (are residue pairs with large couplings on average close in tertiary structure?). We choose a handful of published methods, along with ZNMI, and compare their reproducibility and accuracy on three diverse protein families. We find that (i) of the algorithms tested, while none appear to be *both* highly reproducible and accurate, ZNMI is one of the most accurate by far and (ii) while users should be wary of predictions drawn from a single alignment, considering an ensemble of sub-alignments can help to determine both highly accurate and reproducible couplings. Our cross-validation approach should be of interest both to developers and end users of algorithms that try to detect correlated amino acid substitutions.

## Introduction

With the cost and speed of DNA sequencing improving each year, the number of sequenced proteins is growing much faster than both the number of novel protein families and representative crystal structures. While this sequence redundancy may represent a convergence of knowledge towards the Earth's proteome [1] (with the caveat of possible bias in the niches and organisms that are being sequenced) from the point of view of finding networks of covarying residues in multiple sequence alignments (MSAs) it marks an increase in the number of datasets that can be analyzed. While single-protein investigations (*e.g.* building a small phylogeny or finding conserved sites) require only a modest number of sequences, determining the strength and significance of residue-residue couplings requires many more sequences, with a computational lower limit of 125–150 sequences [2]. The requirement for large sequence numbers has to do with the underlying sources of signal and noise that exist in a multiple sequence alignment (MSA), as observed by Atchley *et al.* [3] (reviewed in [4]). Most users are interested in the part of the signal that results from structural or functional substitutions, but in poorly curated datasets this signal can be masked by the phylogenetic signal [5]. How, then, does one go about assessing correlations in MSAs?

A wide variety of algorithms for detecting correlated amino acid substitutions from a MSA have been developed. Some are based on quantities from information theory [2], [3], [6]–[9], others use chi-squared tests [10], some are perturbative [11], [12], still others employ amino acid substitution matrices [13], [14], and there are many more (reviewed in [4], [15]). Typically, most authors compare their methods against a handful of other methods for a dataset, or in some cases against collections of multiple sequence alignments, such as the Pfam database [16]. While these studies can be illuminating in terms of the novel couplings they reveal and general performance of the algorithms, it is often difficult to compare between them because notions of accuracy and significance vary from author to author. For this reason, a unified framework is needed for comparing and contrasting different algorithms, as well as non-parametric choices that are made.

In some cases, *a priori* constraints are placed on an analysis, for example by (i) a restriction to residues with periodicity of four for 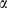-helix interactions [7], (ii) consideration of only specific domain-domain interactions [17], or (iii) a restriction to intraprotein couplings in concatenated alignments [18]. However, these are not general features that may be applied to every analysis as structural information may be unknown, and in a sense they bias the results; one is guaranteed to find domain-domain couplings if *intra*-domain pairs are excluded, but would those *inter*-domain couplings emerge in a more blind approach?

The complexity of determining correlated substitutions has been understated, as it is more than just an issue of selecting the “most accurate” algorithm and proceeding to experimental validation. Another orthogonal feature to accuracy is that of reproducibility or precision: how similar are an algorithm's predictions given different equally informative alignments? This issue has heretofore been completely ignored in the literature. Currently, all coevolving residue studies have assumed a single error-free alignment (*i.e.* statistically, a sample size of one), and thus no information is gained about propagation of errors during the process. The importance of reproducibility is essential if any co-fluctuating networks were to be tested experimentally; mutagenesis of groups of residues followed by tests of fold or function are difficult and laborious, and experimentalists should not waste time testing non-robust (non-reproducible) predictions.

Another area in which the “answer” is produced as a result of a complex, multi-step process with a mix of parametric and nonparametric manipulations is in analyzing medical images, particularly those obtained via functional magnetic resonance imaging (fMRI) [19]. The output of much of fMRI analysis is a statistical parametric map (SPM), a spatially extended statistical model giving information about the regional brain effects of experimental manipulations [20], [21]. The desire to uncover features in the data robust to processing steps and parameter choice has led some investigators to adopt a nonparametric “train-test” statistical approach similar to methods used in machine learning [22]–[24]. The data is split into two groups (split-half resampling [24]) and each group sent independently through the pipeline to produce an SPM. The quality of the data-driven model generated by this data is determined by using the parameters from one SPM to fit the data in the other group (accuracy, measured by cross-validation), and the SPMs are compared between the two groups to find features which are robust to pipeline parameters (reproducibility, usually measured by correlation in the two output SPMs) [23]. Indeed, one can even use this procedure to attempt to optimize the processing pipeline [25]; any equally accurate manipulation which is more reproducible should be adopted in analyzing the data. We take these studies as inspiration and present our own variations on these themes in what follows, in an effort to determine accuracy and reproducibility in the predictions of correlated amino acid substitution algorithms.

In this article, we first introduce a variant of mutual information, called ZNMI (Z-scored-product Normalized Mutual Information) that addresses many of the problems [6], [8], [15] that have plagued mutual information as a metric for predicting coevolving residues (commonly assessed as pairs of residues in tertiary contact [8], [15], though we have more to say on this in “Discussion”). Second and of greater importance, we construct a pipeline sensitivity analysis for testing both the accuracy and reproducibility of coevolving residue detection algorithms. Protein alignments are split into two equal sized sub-alignments and processed identically in order to assess the accuracy and reproducibility of specific algorithms as well as other inherent parameters. Treating the process of determining correlated substitutions as a sequential pipeline in which choices are considered as hyperparameters (*e.g.* how many sequences is enough?, what algorithm should I use?, how should I determine significance?, etc.) in the pipeline allows users to determine the effect of these changes on the resulting accuracy and reproducibility. This can essentially be thought of as a form of statistical cross-validation and a thorough treatment of error propagation when many of the processing steps are nonparametric, not unlike procedures used in machine learning [22].

There is no clear winner among the methods we test in terms of both accuracy and reproducibility, and our results highlight tradeoffs between accuracy and reproducibility, which are bias–variance tradeoffs, as well as dataset-to-dataset variability. Furthermore, the reproducibility of the algorithms tested is very far from ideal and in some cases highly dependent on the dataset analyzed. This suggests that there may be no “one-size-fits-all” correlated amino acid substitution algorithm, or if there is, it is not among the algorithms that we test. Although no algorithm is clearly the best in terms of both accuracy and reproducibility, our resampling procedure provides a unified framework and produces, for any given algorithm, a relatively small number of maximally reproducible disjoint couplings which are close on average in tertiary structure.

## Results

### Adapting mutual information to take into account column variability

Mutual information (MI) [26], [27], a generalization of linear correlation between random variables, has been at the heart of many algorithms for correlated substitution analysis for a number of reasons. Mutual information is naturally defined on symbolic sequences, whereas the application of standard statistical correlations (like Pearson correlation) requires a residue-to-real-number mapping (based on some chemio-physical property or amino acid substitution matrices). In addition, MI has firm theoretical foundations, is relatively easy to calculate as only the individual and joint frequencies of amino acids between columns are needed, and for discrete distributions there is no subtlety in how to bin the values.

Still, MI suffers from a hard to control sources of error, and many authors have pointed out spuriously large MI couplings that aren't likely to be true couplings. Martin *et al.* were the first to use normalized variants of mutual information to correct for bias coming from variable alphabet size among columns [2]. Subsequent work showed that MI suffers from an exceptionally strong linear correlation to the product of the average column mutual information [6], [8]. Together, these two observations imply that pairs of columns with a high MI (i) come from columns with a larger alphabet size and (ii) come from columns which have on average high MI with *all* the other columns in the MSA. Correcting for the alphabet size by normalizing by the joint entropy [2] reduces the correlation, but doesn't entirely remove the bias. Dunn et al. corrected for this bias with a simple multiplicative correction [8], while Little and Chen corrected for this bias via linear regression followed by a two-dimensional z-scoring procedure [6] (see Zres and MIp below).

**Figure 1:**
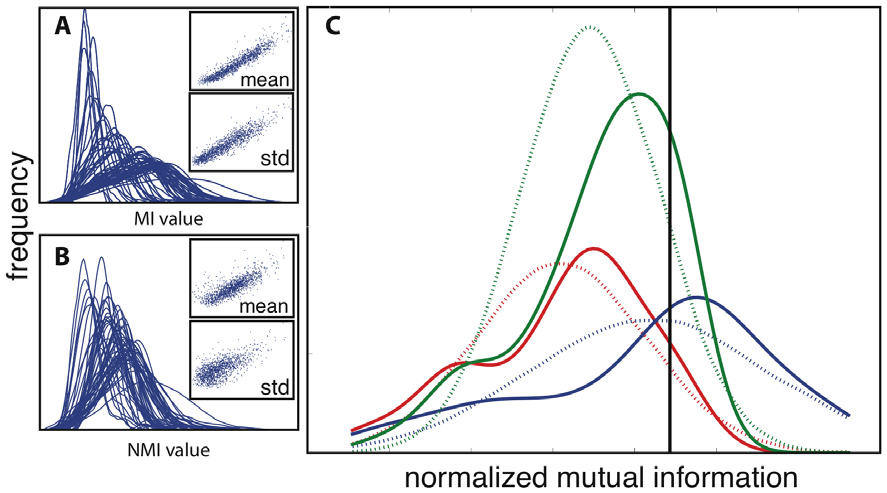
Improving upon mutual information by removing column bias. Mutual information and normalized mutual information is shown for the PDZ dataset. **A.** The distribution of mutual information is shown for each column in the multiple sequence alignment. As can be seen, mutual information is highly correlated to both the product of the mean column mutual information (scatter plot, upper inset) and the product of the standard deviation of column mutual information (scatter plot, lower inset). **B.** The distribution of normalized mutual information (*i.e.* mutual information normalized by joint entropy) is shown for each column in the multiple sequence alignment. The normalization reduces both the correlation between the product of the mean column mutual information (scatter plot, upper inset) and the product of the standard deviation of column mutual information (scatter plot, lower inset), but doesn't remove it entirely. **C.** ZNMI approximates the column normalized MI distributions (solid red line and solid blue line) as Gaussian distributions (dashed red line and dashed blue line), calculates a closed-form expression for the product of the two distributions (solid green line: kernel density estimate of product), and then z-scores the normalized mutual information (black solid vertical line) based on the Gaussian approximation of the product (dashed green line).

In order to address these bias issues in a straightforward way, we introduce a variant of MI known as ZNMI (see “Methods” and Figure 1). Given that mutual information is highly correlated to the product of the average column mutual information (Pearson's 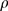 = 0.97, Figure 1A), we also asked whether mutual information is linearly correlated to the product of higher moments of column mutual information. Striking correlation (Pearson's 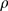 = 0.96, Figure 1A) does exist between the MI and product of the standard deviations of the column mutual information; hence columns whose average MI is larger *and* more widely distributed tend to end up with a high MI when paired. Normalizing by the joint entropy reduces this correlation, but does not remove it (Figure 1B). In an effort to further remove this bias, we approximate the column normalized mutual information distributions as Gaussian distributions (Figure 1C). Because Gaussian distributions are closed under products, the product of these two distributions is again a Gaussian distribution (see “Methods”). We use this product distribution to calculate a z-score for the normalized mutual information originating from the original two columns. This treatment amounts to asking: how significant is the normalized mutual information between two columns given the background normalized mutual information column distributions? A pair which is an outlier in MI should be in the tails of *both* column distributions, and our procedure takes into account the width of both tails.

### Datasets and pipeline

**Figure 2:**
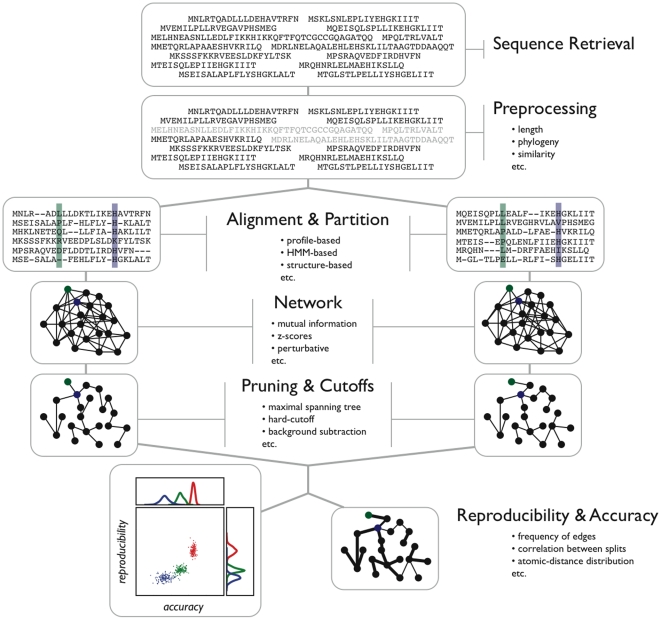
Overview of the statistical pipeline. Determining intra/inter-protein coevolving residues can be thought of as a complex, mulit-step optimization process. Initial sequences, as many as possible, are collected for a protein of interest (**Sequence Retrieval**). The sequences are pruned by similarity and length in order to filter the starting dataset of sequence fragments and sequences that heavily bias the phylogeny (**Preprocessing**). The sequences are then aligned by available methods, and many independent disjoint splits of the dataset are made so that half of the aligned sequences are in one split and the other half are in the other split (**Alignment & Partition**). From this point on the two splits of the data are processed equivalently. A coevolving residue algorithm is then used to convert a split of the data (sub-alignment) into a correlation matrix that can be analyzed as an undirected weighted graph (**Network**). The resulting graph can then be pruned to remove insignificant edges or highly gapped columns (**Pruning & Cutoffs**). Finally, the independent splits are compared and result in measures of accuracy and reproducibility (**Reproducibility & Accuracy**).

We chose three diverse protein families to study: chorismate synthases (CS), G-protein coupled receptors (GPCR), and the PDZ domain (PDZ) [28]–[30] (see “Methods”). All three of these datasets have been the focus of other correlated substitution studies [6], [9], [11], [17], [31]–[33]. We investigate each dataset in our statistical pipeline and tweak various parameters. Figure 2 shows a flow diagram of the generalized steps in our analysis pipeline; a full description of each step as it was implemented by the authors can be found in “Methods.” What follows is a general summary of the pipeline framework.

Sequence selection and preprocessing are the initial two steps. Following this, sequences are aligned and partitioned many times into two disjoint sets (a 2-split); each partition contains half of the sequences in the full alignment. For a given 2-split, pair scoring methods are computed for each subalignment and the results visualized as an undirected, dense, weighted graph in which residues are nodes and edge weights between nodes correspond to the pair score. The resulting dense graphs are pruned and are subsequently compared to obtain measures of accuracy and reproducibility. By considering all 2-splits, we can construct a consensus network whose edge weights correspond to the number of times (or frequency) that edge was present in a subalignment's pruned graph. This cross-validation scheme involving 2-splits of the MSAs yields measures of accuracy and reproducibility that can be compared between different datasets, across different procedures.

### Pipeline sensitivity

#### Scoring method comparison

**Figure 3:**
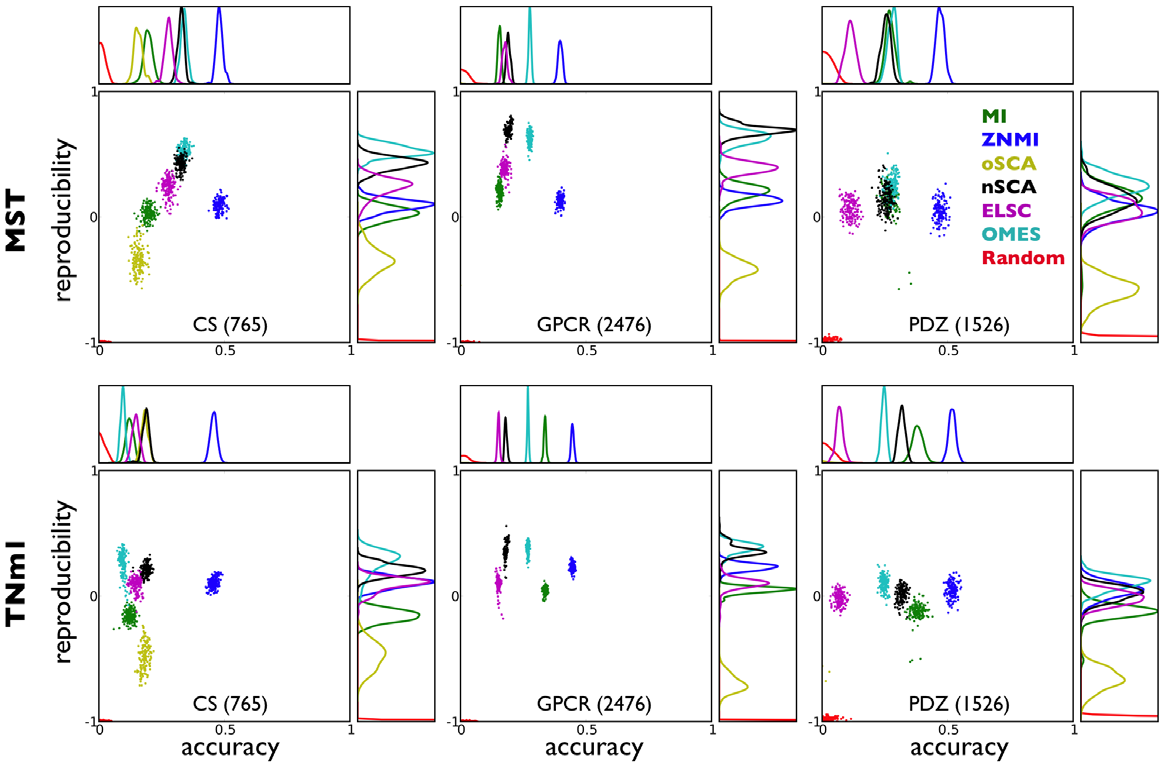
Reproducibilty and accuracy for published algorithms on three different families. Scatterplots and histograms of reproducibility and accuracy for the three protein families (PDZ, 1256 sequences, CS, 765 sequences, GPCR, 2476 sequences) we consider in the text. The methods are Random (red), MI (green), old SCA (yellow), new SCA (black), OMES (cyan), ELSC (magenta), and ZNMI (blue). The top row shows the results when we construct the consensus network using MST, and the bottom with TNm1. The y axes on the reproducibility histograms have been rescaled to allow better visualization of the shapes of the distributions. The line colors shown in the GPCR MST panel are used consistently throughout. The old version of SCA often produces accuracies below that of random (near zero, left side of each plot); see the text for further discussion on this point.

Figure 3 shows reproducibilityand accuracy results for the CS, PDZ, and GPCR protein families for many different scoring methods (see “Methods”). We show the results of constructing the consensus network via both maximal spanning trees (MST) and simply selecting the largest scoring 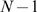 edges (TNm1) (Figure S1). Although in this paper we force all algorithms to make roughly N predictions (for comparison reasons), this overlooks an important point about thresholding. Generally, each algorithm will make a different number of statistically significant predictions and a proper threshold should be established for subsequent reproducibility and accuracy calculations (see “Discussion” for more details). Still, algorithm performance is extremely consistent over both consensus network construction methods and protein family. We first notice that Rand always performs extremely poorly at finding residues close in tertiary structure, and is utterly irreproducible, as we expect. Surprisingly, oSCA, while more reproducible than Rand, is typically (in four of the six panels in Figure 3) *less* accurate, indicating that it tends to assign high scores to pairs of residues which are further apart in tertiary sequence than if they were picked at random.

Another consistency is the performance of ZNMI. ZNMI is consistently one of the most accurate methods, and never fares very poorly in terms of reproducibility. However, the most reproducible algorithm is almost always OMES, though nSCA is usually quite close. We also wondered whether other newer MI-based algorithms that try to improve upon the performance of MI, namely MIp [8] and Zres [6], perform similarly to ZNMI. As can be seen in Figure S2, while ZNMI and MIp preform similarly for the three datasets, Zres outperforms both algorithms for two of the three datasets (excepting the GPCR dataset). Taken all together, a tradeoff is seen between highly accurate algorithms, such as ZNMI and Zres, and highly reproducible algorithms, such as OMES.

These analyses highlight an important message: *reliable calculations of co-fluctuating networks of residues from multiple sequence alignments may introduce a reproducibility/accuracy tradeoff in addition to dataset-to-dataset variability, and there may be no* “*one-size-fits-all*” *method*. We don't know the conversion or tradeoff between accuracy and reproducibility, known as the reproducibility-accuracy Pareto surface or frontier in optimization theory [34], and consequently cannot declare a clear methodological winner for the GPCR dataset. For the other two datasets, PDZ and CS, we find tradeoffs between accuracy and reproducibility between most methods, with the exception of Zres, which seems to be the clear winner (Figure 3, Figure S2).

A more accurate method could simply be finding residues closer in *linear* sequence, thus guaranteeing their proximity in tertiary structure. A simple example of this would be for an algorithm to return nearest-neighbors in linear sequence. This would result in trivially “close” residues in tertiary structure. In order to rule-out this trivial determinant of accuracy, we calculated the average linear sequence separation versus accuracy for each of the datasets. While for the PDZ dataset, increasing accuracy does mean a decline in the average linear sequence separation, for both the GPCR and CS datasets linear sequence separation for all methods (except Rand) varies by 10–15% but accuracy can be increased by up to a factor of 5 by using ZNMI (Figure S3). Even for the PDZ dataset, one can gain a factor of 2 in accuracy over OMES or nSCA while only being on average 4 residues closer in linear sequence (Figure S3).

#### Effects of sequence selection and alignment method

One expects that both accuracy and reproducibility should increase as more informative sequences are added to the alignments. In order to check that this is the case, we used three nested subsets of sequences for each of the three protein families and calculated the resulting reproducibility and accuracy (see “Methods”). Consistent with what one would expect, increasing the number of informative sequences does increase the resulting reproducibility and accuracy for all three datasets (Figure S4). There is a subtle caveat with respect to the concept of just how “informative” a sequence is: because sequence conservation can stem from two extremes (*i.e.* conservation amongst phylogenetically distinct sequences or merely redundancy due to phylogenetic/sampling bias), the sensitivity tools we present here are not completely adequate to decide whether an initial dataset is optimized. Although this issue has only been touched upon in the literature [4], we feel it is an important open question and leave it as a future research direction (see “Discussion”).

A final parameter to investigate is the influence of different alignment methods. Figure S5 shows the influence of using two different alignment methods (MUSCLE [35] and MAFFT [36]) on the resulting accuracy and reproducibility. A quick comparison of the scatterplots for these three datasets shows that the choice of alignment method has little affect on the resulting accuracy and reproducibility for any of the methods. This is not to say that one shouldn't take care in curating a good starting alignment. Although the resulting accuracy and reproducibility remain almost invariant, it is not the case that each alignment method leads to the exact same edges in the consensus network; the Jaccard index (see “Methods”) is less than 1 even at a very high frequency cutoff in the consensus network (data not shown). This behavior can easily be explained by the fact that the canonical sequence (*i.e.* the sequence that is used for numbering the final graphs) is slightly perturbed between the two different alignments, and thus edges with slightly different nodes (off by one or two in linear sequence) are present.

#### Effects of network pruning

While the reproducibility and accuracy results are similar for consensus network construction via MST and TNm1 (Figure 3), we wondered whether a pruning step is imperative (*i.e.* are the lowest scoring couplings as reproducible and accurate as the top ones or are they generally noisy and inaccurate?). To investigate this we calculated the reproducibility and accuracy by selecting the *bottom* 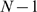 scoring couplings (BNm1) to construct the consensus network (Figure S6). Notice that all methods suffer a huge penalty in accuracy, confirming as one suspects that the weakest couplings are essentially noise. Not only are these weak couplings inaccurate, they are generally irreproducible, which can be seen by comparing to Figure 3. Interestingly, oSCA is *more* reproducible for the GPCR dataset when selecting the lowest scoring edges than when selecting the highest scoring edges; this combined with its odd accuracy behavior in Figure 3 suggest that oSCA is not a promising method, perhaps leading to the development of nSCA.

#### Consensus network as a function of cutoff

**Figure 4:**
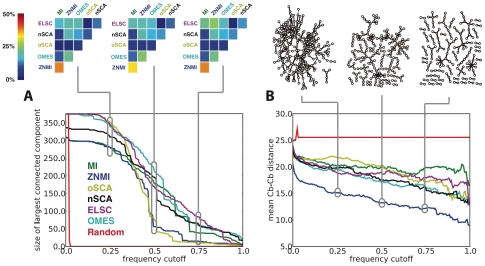
Weights in the consensus networks decay dramatically. **A.** The largest connected component in the MST consensus network, for the full CS dataset, as a function of edge weight cutoff is shown. For all of the edge scoring methods considered, but particularly ZNMI and oSCA, use of MSTs to construct the consensus network results in small, disconnected clusters when the consensus network is relatively mildly pruned. Directly above the plot, heatmaps are displayed for the Jaccard index (all methods vs. all methods, excluding Rand) for three points along the curve (0.25, 0.5, and 0.75). As the network is pruned, the Jaccard indices generally remain the same with only slight increases in overlap between methods (note: ZNMI and ELSC at a cutoff of 0.75). Note that the colorscale is given not in terms of the actual Jaccard index but the percent similarity between the two sets of edges (see “Methods”). **B.** Cutting the graph with increasing edge weight results in edges that are in fact closer in tertiary structure, as measured by their mean 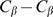 distance. Directly above the plot, the consensus graph is shown at three different edge frequency cutoffs. Note the dramatic transition in the consensus graph between a weight of 0.25 and 0.5; simply removing edges which co-occur less than 50% of the time results in a network consisting primary of small, disjoint clusters. Notice also that even at a cutoff of 0.75, many nontrivial clusters (beyond simple pairs) remain in the network.

The consensus network calculated by any of the methods we have described (MST, TNm1, BNm1) is a weighted graph; each edge has a weight equal to its frequency of occurrence during the resampling procedure (*e.g.* if an edge appeared in 240 of the 300 graphs (resulting from 150 splits), then it would have a weight of 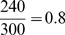). Figure 4 shows the largest connected component of the consensus network (Figure 4A) and the mean tertiary distance of the predictions (Figure 4B), as a function of pruning by increasing edge weight. For three pruning values (0.25, 0.5, and 0.75), additional information is provided above the plots.

As Figure 4A shows, a steep decline in the size of the largest subgraph component is seen for all methods, but especially for ZNMI and oSCA. Above, Jaccard index heatmaps compare the overlap in predicted edges for all pairwise method comparisons (see “Methods”). Several features of these heatmaps are of note. First, no two methods produce terribly similar consensus networks, at least when considering all edges. The overall degree of inter-method similarity rises marginally as the least robust edges are removed; the heatmaps aren't becoming substantially more yellow-red as the cutoff is increased, except for a few instances. Also, the two most similar methods are MI and ZNMI, which is expected given that ZNMI has MI at its core. Figure 4B shows that edges of higher frequency (*i.e.* more reproducible) are close in tertiary structure, so that pruning the consensus graph at a higher cutoff results in more residues proximal in tertiary structure, as measured by their mean 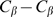 distance. While all methods with exception of Rand are monotonically decreasing functions of frequency cutoff (with respect to mean 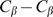 distance), ZNMI performs best. Above, the consensus network produced for the CS dataset by ZNMI at cutoffs of 0.25, 0.5, and 0.75 are shown. Simply using a frequency cutoff of 0.5 versus 0.25 vastly simplifies the resulting co-fluctuating residue networks, and truncating to edges that only occur in 75% or more of the splits results in primarily isolated couplets of residues with a few larger groups (upper right panel; recapitulated in the size of the largest connected component in the lower left panel). However, even at a stringent 0.75 cutoff there are co-fluctuating residue networks with nontrivial structure; they are not simply pairs. Still, as Figure 4B shows these edge weights decay dramatically; the number of robust edges (those with a weight near unity) is a very small fraction of the total number of edges in the dense consensus graph.

### Clusters mapped to structures

**Figure 5:**
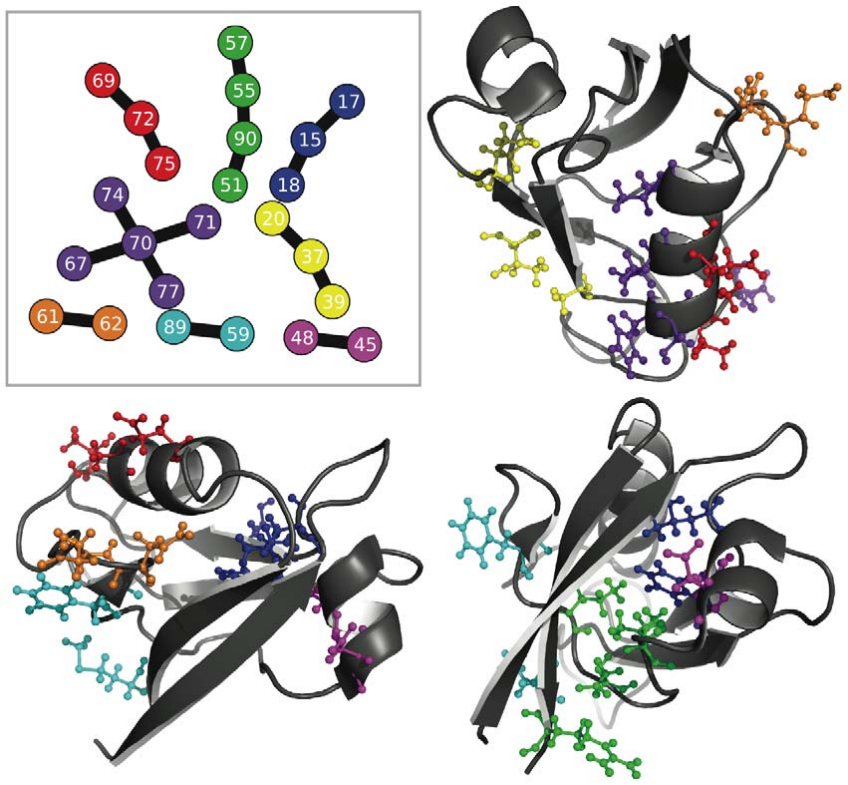
Consensus communities at 90% reproducibility mapped to the PDZ tertiary structure. The upper left panel shows the consensus network for the PDZ dataset at a reproducibility cutoff of 90%. The remaining three panels give three views of the consensus networks mapped to our chosen canonical PDZ structure (PDB Identifier: 1IU0). The color coding in the upper left panel is identical when considering the structures. While some of the consensus co-fluctuating groups are quite close in sequence (orange and dark blue), others (cyan) are quite far away. A closer look at the red and dark purple clusters is given in Figure 6. For this figure and Figures 6–10, ZNMI is the pair scoring method and MSTs were used to construct the consensus networks.

**Figure 6:**
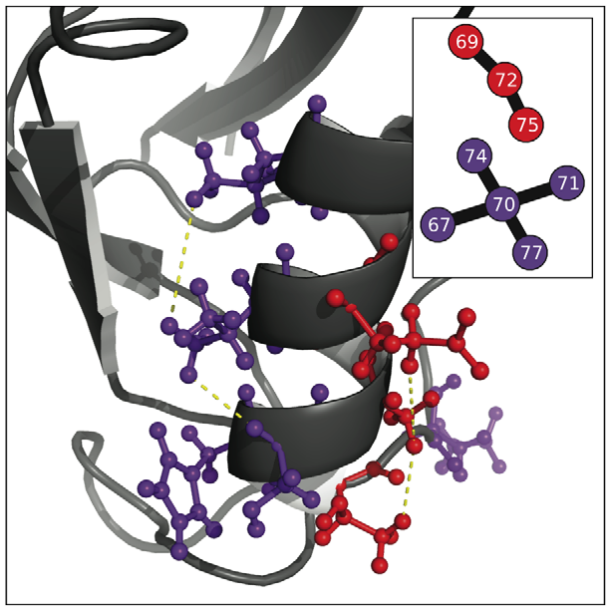
Two disjoint but intertwined communties mapped to the PDZ tertiary structure. Shown here is a closeup of the red and purple clusters from Figure 5. These two communities are disjoint at this cutoff (90%) and on opposite sides of the pictured helix. Also of note is that they have a periodicity of three in sequence, not four residues as would be the case with residues interacting through the turns of an 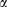-helix.

**Figure 7 pone-0010779-g007:**
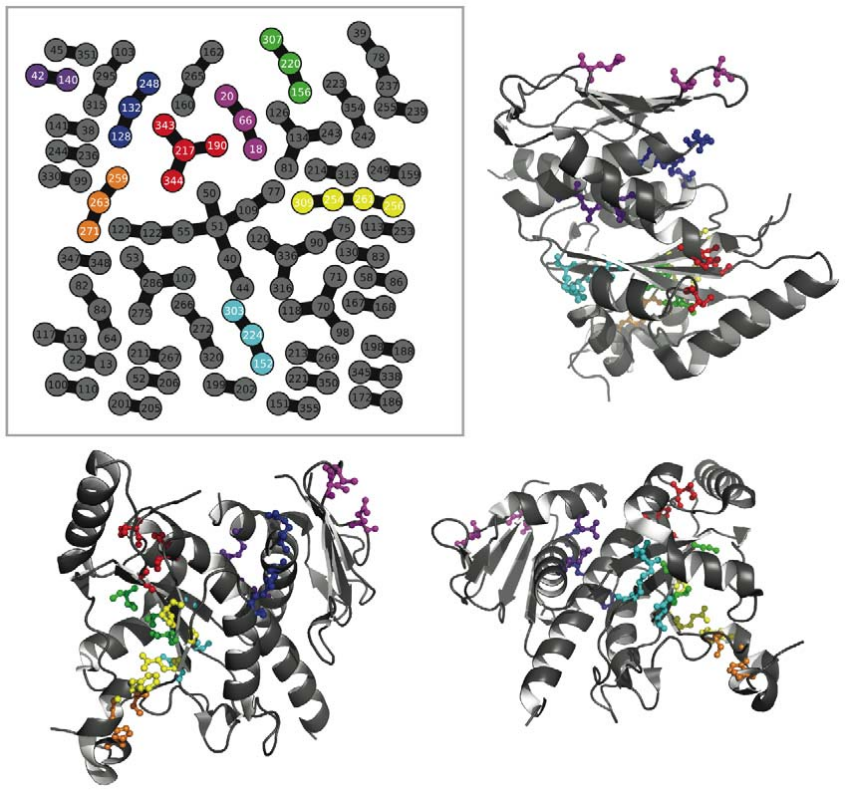
Consensus communities at 90% reproducibility mapped to the CS tertiary structure. The upper left panel shows the consensus network for the CS dataset, again at a reproducibility cutoff of 90%. Several of these communities have been colored in and mapped to the canonical structure (PDB Identifier: 1R52); the color code is consistent between the networks and the structural views. The magenta community is considered more closely in Figure 8.

**Figure 8 pone-0010779-g008:**
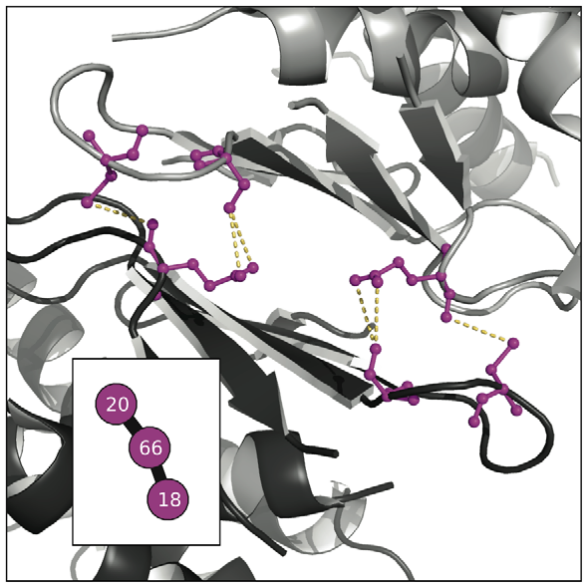
Small community in the CS consensus network highlights a dimerization interface. Here we show a closeup view of the CS structure from Figure 7 and the network colored in magenta. Viewed on a single copy of the CS structure, the magenta community seems to be meaningless. However, when CS dimerization is considered, the magenta community shows its role as a key set of residues mitigating an inter-subunit coupling. Also of note is that the residue topology in the consensus network exactly mimics their minimum distance topology in the tertiary structure.

**Figure 9 pone-0010779-g009:**
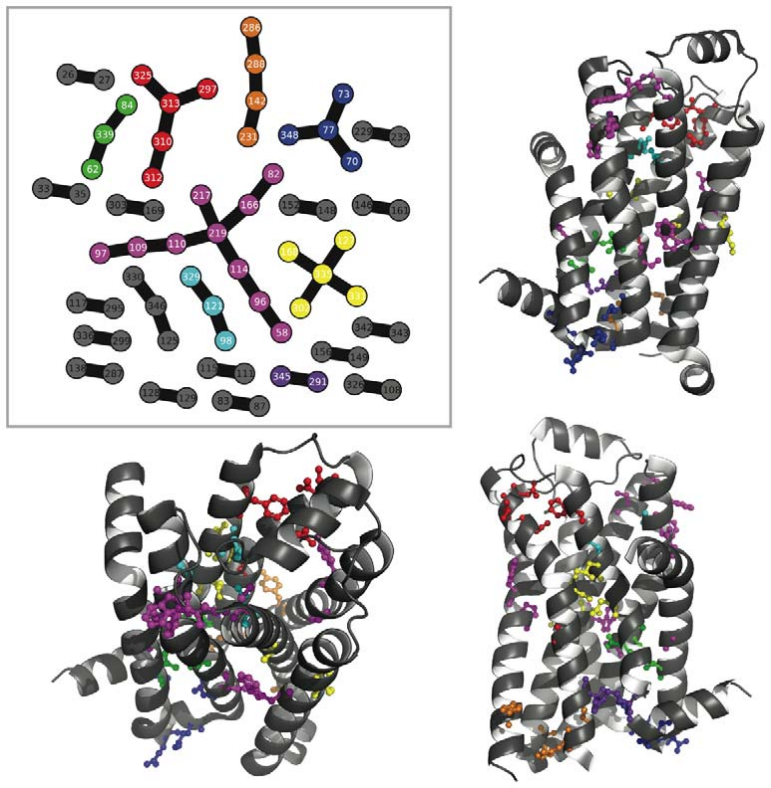
Consensus communities at 90% reproducibility mapped to the GPCR tertiary structure. The upper left panel shows the consensus network for the GPCR dataset at 90% reproducibility, and the remaining panels show selected communities mapped onto the canonical structure (PDB Identifier: 2VT4). The consensus network here was computed from a 1000-sequence GPCR dataset because it was more accurate than the full 2476 sequences (an average of 10 angstroms vs 15 angstroms).

**Figure 10:**
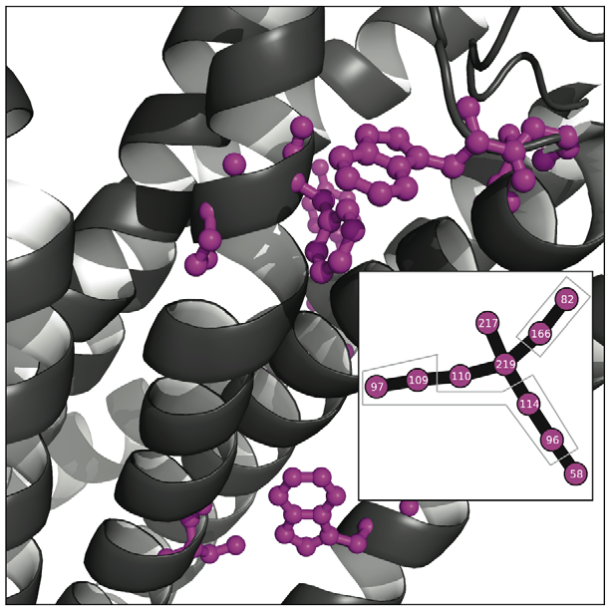
One large community from the consensus network at 90% reproducibility mapped to the GPCR tertiary structure. We show here an enlargement of the magenta community from Figure 9. The inset shows the cluster along with two boxes highlighting two portions of the community. Note that this cluster shows significant coupling at large physical distances; the four residues outlined in the inset are at the top of the figure and the other two outlined residues are at bottom.

Figures 5, 6, 7, 8, 9 and 10 show the most reproducible clusters of co-fluctuating residues mapped to the corresponding canonical structures for the PDZ, CS, and GPCR datasets. For all of these figures, we show only couplings present at a reproducibility criterion of 90% or greater as calculated from the ZNMI algorithm; that is, a link has to be present in 90% or more of the subalignment MSTs. For most of the communities we show, the residues appear to be in tertiary contact and the likelihood that they represent real interactions, either functional or structural, seems quite good. Note, for example, that for the GPCRs (Figure 9) we find many clusters that represent interactions between helices in the seven-helix transmembrane spanning barrel, despite *not* having restricted the analysis to only pairs between these helices, as has been done previously [17].

#### PDZ

Figures 6,8,and 10 show particularly interesting communities from each protein family in detail. Although the PDZ domain is a relatively small protein, interesting communities are present. Figure 6 shows two communities in the PDZ network which are disjoint at the 90% reproducibility level but which intertwine. They are on opposite sides of the same 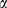-helix and have an almost perfect periodicity of three residues in sequence, contrary to the expected periodicity of four one would find for residues interacting through the turns of a helix.

#### Chorismate Synthase

Figure 8 shows a highly reproducible community in the chorismate synthase family that is likely relevant for the function of proteins in the family. Considered as a monomer, the magenta community (shown first in Figure 7) looks cryptic but when dimerization is pictured the cluster assumes an immediate significance as part of the dimerization interface. Viewed properly this way, the cluster's topology even mimics the distance topology one obtains when looking at the structure. Chorismate synthase has been widely found to be active as a dimer or tetramer in bacteria [37], [38], fungi [37], and plants [39]. CS is part of a pathway producing aromatic amino acids in these organisms. The fact that mammals lack this pathway and obtain tryptophan, tyrosine, and phenylalanine via their diets has led to the suggestion that CS and the shikimate pathway in general would make a good antibiotic target [38]. Disrupting the co-fluctuating cluster in Figure 8 could accomplish this in a wide variety of organisms, given that it came not from a single protein structure but from a MSA. This points to the potential of using correlated amino acid substitution detection for therapeutic intervention. Also, we emphasize that this cluster was present in almost all subalignments; it is one of the most robust signals in the CS dataset.

#### G-Protein Coupled Receptors

Figure 10 displays an interesting co-fluctuating cluster in the GPCR dataset. Two segments of the cluster have been outlined in grey; the group of four residues near the top of the picture and the pair that are quite far away from the top four residues. Within the groups the residues are in close contact in the tertiary structure, but notice that between the groups there is a substantial space spanned in tertiary structure. This result highlights an ongoing debate in the literature about the length scale over which residue–residue couplings would interact, especially with respect to allostery. Are long-range interactions mediated through couplings at a distance, are there networks of simple pairwise interactions that mediate communication at great distances, or are these couplings simply biologically-meaningless false positives [9]–[11], [15], [32], [33]? In the case of the GPCR dataset, we don't find a large reproducible community (at the level of 90% frequency) that spans the entire protein from the allosteric site to the intracellular G-protein coupled site; the largest cluster shown in magenta is quite dispersed throughout the protein, and the remaining clusters are small and localized. This is consistent with an ensemble-based explanation of allostery that involves perturbations to the population of energetic states around the native state, and not the existence of intricate pairwise-coupling pathways or sequential conformational changes [40]. We interpret these results (as well as the results for the preceding datasets) in two general ways. Many of the networks that these algorithms find are presumably important for folding (rather than function) and folding is believed to be a process of local condensation rather than global collapse encoded by the native state [41]. For this reason, we feel that many of the small (composed of two to five residues) clusters may be important for folding. Furthermore, as the community in Figure 10 may suggest, residues at a distance may be coupled due to the inherent dynamic nature of a protein undergoing conformational changes that aren't foreseeable in a single crystal structure.

## Discussion

We have presented an improvement to mutual information for use in correlated amino acid substitution analysis. More importantly, we have cast the problem in a framework that allows a “meta-analysis” of any method, and all parameters of those methods, that simultaneously ranks algorithms on two criteria: accuracy, defined here as closeness in tertiary structure, and reproducibility, defined as sample-to-sample consistency. This allows one to consider new algorithms and adjustment of algorithmic parameters in an optimization framework; the goal is simultaneous optimization of both. We hope that this will be of interest to both methodologists and end users; methodologists can test a new algorithm in this framework, and end users can obtain some idea as to the confidence they should place in a cluster. One would use datasets for which a canonical structure exists (such as the three in this manuscript) to gain some idea of method quality, and then apply the best method to their own dataset of interest, possibly without structural information.

There are two highly desirable extensions to this study that are at present unattainable. Those are (i) using our pipeline sensitivity process to guide sequence selection itself and (ii) assessing the utility of these algorithms for predictions of residue coevolution (testing the so-called “covarion hypothesis”), as completely distinct from contact prediction. Both of these studies would be relatively straightforward but both are at present intractable. One would like to use the reproducibility/accuracy metrics in a “meta-optimization” that not only yields robust predictions *given* the input data, but also helps to select that input data in order to jointly maximize reproducibility and accuracy. For example, simply having many redundant sequences rather than fewer diverse sequences is likely to negatively impact contact prediction, and one would like to choose the optimal input alignment for this process. The computational barriers to doing this for all but the smallest protein fragments make this prohibitive; however as a thought experiment, it seems to clearly be the correct thing to do.

As for (ii), we have used distance in tertiary structure as our accuracy metric. Some would argue that this is inappropriate [11], [32] and that residues very far apart in tertiary structure can be coupled as strongly, or more strongly, than those nearby; yet others would disagree [15]. We point out that our accuracy metric is an *average*, and may have wide dispersion. A method with high accuracy need not, and generally will not, entirely exclude residue-residue couplings which are far apart in tertiary structure; indeed, Figure 10 shows that ZNMI finds a clear signal of a cluster with coupling at large physical distance. While couplings at large distances could still simply be false positives, for them to appear as robust predictions in our meta-analysis they must occur as relatively large signals in practically all the splits of the data. This does not rule out the false positives, but it makes it somewhat harder to believe. If alignment errors produce them, they are alignment errors that recur in a large fraction of the subalignments. In any case, the significance of strong long range couplings detected by correlated amino acid substitution analysis will likely remain unresolved without an experimentally validated “co-evolutionary” dataset. If we knew which networks of residues most strongly fluctuate during evolution, even for only a single protein or protein family, we could use the resampling framework presented in this manuscript to determine which algorithms robustly predict those coevolutionary networks. However, lacking such a dataset, the only way to validate algorithms predicting co-fluctuating positions is to use as training sets protein families for which structural information exists.

An interesting feature of our results is the “no-man's land” in the plots in Figure 3, namely the upper right corner of the scatterplot. An algorithm whose scores fall in that area would be both highly reproducible and highly accurate, and none of the methods we consider here reach that level of performance, irrespective of the dataset in question. Therefore, it is unwise to simply run the algorithms investigated here only once on a single alignment. Despite the lack of a highly reproducible and accurate algorithm, the resampling framework presented here can associate a confidence (*i.e.* the frequency cutoff in the consensus network) with individual couplings. While tradeoffs between reproducibility and accuracy are inevitable, especially with small-to-moderate sample sizes as one finds in realistic datasets [24], that does not rule out pushing the boundary of algorithm performance further into that quadrant. We only expect that at some point we will be forced to trade bias for variance, but we do not know where that frontier is or if we have reached it [24].

One potential issue that has been overlooked in our framework is the issue of thresholding. In general, the number of edges should not *a priori* be fixed (*i.e.* a MST fixes the number of edges to 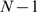). Each algorithm will produce a different number of statistically significant couplings, and proper thresholds should be established individually for each algorithm. We did not investigate this (for comparison reasons), but instead bring it to the reader's attention and leave it as a future research direction. Although each algorithm will require a specific thresholding scheme, ZNMI allows for a clever thresholding scheme simply by its construction (kindly pointed out by an anonymous reviewer). Because each normalized mutual information value is compared against a background Gaussian expectation, then a p-value can be associated with each column pair. Subsequently, the p-values could be corrected for multiple hypothesis testing with a simple Bonferroni correction. Still, the idea of setting appropriate thresholds and combining methods (*e.g.* combining Zres, a MI-based metric, with OMES, a non-MI based metric) into a “meta-method” further point out the machine-learning possibilities of our framework, and we are actively exploring these avenues.

## Methods

### Processing Pipeline

Figure 2 gives a schematic describing the steps in the processing pipeline leading to predictions of co-fluctuating residue groups. We consider each step in more detail below; some steps (like the scoring algorithms used) are also described in much greater detail elsewhere in the methods section. We emphasize here that many of the hyperparameters in the analysis are nonparametric, often amounting to “do 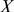 or 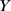
” or “do 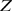 or not 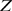,” making a cross-validation scheme the most effective way to understand the propagation of errors during the calculations.

#### Sequence Retrieval

We chose three diverse protein families to study: chorismate synthases (CS), G-protein coupled receptors (GPCR), and the PDZ domain (PDZ) [28]–[30] (see “Datasets” in this section for more details). All three of these datasets have been the focus of other correlated substitution studies [6], [11], [17], [31]–[33].

#### Preprocessing

Before analysis, we pruned the sequences to remove fragments and redundant sequences [4]. It is important to point out the effect of this filtering on the PDZ and CS datasets, which were retrieved from the Pfam database [16]. In both datasets, the initial number of sequences is around 5000, but after pruning the datasets are significantly smaller, with less than one-third of the sequences retained in the PDZ dataset and a mere one-sixth retained in the CS dataset. While some fragmented sequences are removed, which helps with alignment performance by limiting the number of gaps in the MSA [4], the majority of the removed sequences are simply highly similar (greater than 95%) and therefore redundant. This is important to point out as it is not a universal practice to remove redundant sequences. Many authors use the curated alignments from the Pfam database without parsing for redundancy; this redundancy is more harmful than it seems as it can drastically alter the frequencies of amino acids and the subsequent couplings between them.

#### Alignment and Partition

Sequences are aligned and many 2-splits are made, such that for a given split each of the two resulting groups contains the same number of sequences and each group contains the same canonical sequence (used for numbering and structural mapping). There are many alignment methods available that differ in the amount of *a priori* information (including structural) they employ, computational complexity, etc.: MUSCLE [35], MAFFT [36], HMMER [42], and T-Coffee [43] are four we have investigated. We used MUSCLE for all of the alignments in this manuscript, except for those compared in Figure S5, where we investigate the influence of alignment method. For this comparison, we also made alignments using MAFFT. For each of the datasets in this study, 150 independent partitions were made for calculations of accuracy and reproducibility.

#### Network Construction

Correlated amino acid substitution scoring metrics produce a set of real numbers, one for each pair of residues in the (canonical) sequence. This matrix of values can naturally be viewed as a weighted graph, in which the nodes are residues and the links between the residues are assigned weights according to the results of the pair analysis. Before implementing a more complicated pruning method (see Network Pruning below), we first remove those nodes that are more than 10% gapped in the MSA. For numerical reasons, we also remove any nodes whose column entropy in the MSA isn't greater than 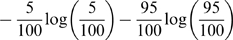, which ensures that enough of the residues in a site are changing to measure a co-fluctuation (*i.e.* 5%). Finally, we remove any nodes for which the canonical sequence is gapped.

#### Network Pruning

When pruning a network, one would then like to pull out groups of residues more strongly connected to each other than to the rest of the residues in the protein, as is the goal of all so-called “community detection” algorithms for networks [44], [45]. Unfortunately, the graphs resulting from co-fluctuating residue analysis are (i) extremely dense (each residue is connected to every other residue) (ii) weighted graphs, in which (iii) the dynamic range of the weights is modest. These features make existing community detection algorithms of little use; our attempts to find communities by maximizing Newman's modularity [46] were fruitless (not shown). In addition, graph segmentation algorithms are generally complex optimization procedures in which little information about community robustness is accessible.

The complexity of coevolving residue networks leads most authors to prefer some sort of “top hit” analysis, in which some (often arbitrary) number or percentage of top scoring residue pairs are selected as the most reliably predicted co-fluctuating groups. We also prune the dense graphs, but our pipeline sensitivity calculations allow us to compare different pruning methods. We generally use two methods: in one, we retain the maximal spanning tree (MST) of the full scoring graph. The MST for a graph with 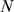 nodes is an acyclic connected graph with 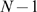 edges; each of the 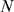 residues in the protein will be present in the MST, assuming they aren't heavily gapped positions in a MSA (see Network Construction above). We also simply keep the top scoring 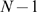 edges (TNm1), and sometimes the lowest scoring 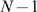 edges for comparison (BNm1). An example of both an MST and a TNm1 graph for a single subalignment of one protein family is shown in Figure S1.

#### Reproducibility and Accuracy

We define the reproducibility for a split as follows. For each split, two pruned graphs are calculated - be they MSTs, TNm1s, or BNm1s (the two graphs are denoted below as set A and set B). We then compute the Pearson correlation coefficient of the edges of the two graphs. Edges in the intersection are counted in the correlation using their weight, and edges in one graph but not the other are assigned a weight of zero in the graph in which they are not present. We should point out that, using this definition, it is easy to obtain a negative reproducibilty, which simply means that the set of intersected edges is small relative to the total number chosen. We employ this definition, rather than restricting the correlation to only shared edges, both because it maintains a sensible scale for the reproducibility (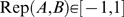) and because it allows us to compare the value across splits and algorithms, as the number of data points used in calculating 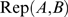 remains constant whenever the same number of edges are retained at the pruning step. A negative reproducibility should cause no concern; we are simply concerned with *increasing* reproducibility and not its magnitude.

Ideally, a measure of accuracy for algorithms that predict coevolving residues would measure deviations from a validated dataset, just as some data is reserved in machine learning problems in order to train a classification or regression algorithm. Unfortunately, no such dataset currently exists, and it is unclear if one can be easily and meaningfully generated. However, if we view this as a contact prediction problem, we can define the accuracy as the average proximity in tertiary structure of nodes connected by edges, weighted by the strength of the edge. These distances are calculated using 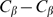 distances obtained from the canonical structure. For a given split, the accuracy is defined as
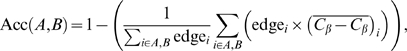
(1)where
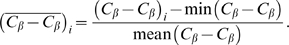
(2)Intuitively, Eqn. 1 is just reversing low values and mapping maximal accuracy as 1, with the term inside the parentheses being nothing more than a weighted average (*i.e.* algorithms that assign large weights to residue pairs that are close in tertiary structure result in a lower weighted average and higher accuracy). Eqn. 2 is rescaling the residue–residue distances by the minimal attainable value and the average value (*i.e.* the value that an algorithm would achieve blindly picking residue pairs). Overall, the definition of accuracy sets a baseline of zero accuracy for the Rand algorithm, with a maximal achievable accuracy of 1. Note: we are only rescaling accuracies between 0 and 1. Accuracy can be negative, as is the case with oSCA in 4 our of 6 panels of Figure 3, but we aren't concerned with negative accuracies and thus algorithms that on average perform worse than random selection of residue pairs.

We emphasize that reproducibilty and accuracy can be completely independent; one can easily construct a perfectly reproducible “method” (pick the same pairs always, regardless of scoring metric) that is as inaccurate as possible (pick the pairs furthest apart in tertiary space). We also emphasize that these are the definitions of accuracy and reproducibility that we chose to implement. These definitions can be altered to suit an end user's needs. For example, choosing a metric of reproducibility that uses the intersection of splits containing differing set sizes (i.e. Fisher transformed correlation coefficient), or a measure of accuracy that assigns a binary classification to tertiary distance (i.e. CASP prediction criteria) are alternative definitions and can thus be investigated in our pipeline framework. We have chosen not to use these alternative definitions as they introduce additional complexity. For example, comparing correlation coefficients of datasets containing a different number of points (via a Fisher transform) loses its correlation-type interpretation of reproducibility. Similarly, allowing for a binary classification of accuracy introduces yet another hyperparameter into the pipeline (*i.e.* the cutoff used for the classification), which would need to be investigated.

### Datasets

Sequence datasets were downloaded and processed as described below. Calculations of sequence similarity and the informativeness of sequences was done using the T-Coffee package [43]. The number of sequences remaining after each step of the preprocessing is indicated in parentheses. The canonical sequence used for mapping the residue positions to the tertiary structure is indicated for each dataset by its PDB identifier. For the smaller nested datasets used in Figure S3, the 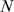-most informative sequences were extracted from the next largest dataset (*e.g.* for the CS dataset, the 200 most informative sequences were extracted from the 400 sequence dataset) using the T-Coffee package [43] and keeping the canonical sequence.

#### CS

Chorismate synthase Uniprot and NCBI headers were extracted from Pfam entry PF01264 datasets, PF01264.full and PF01264.NCBI, respectively [16]. The full sequences were retrieved from NCBI (4198 sequences) and Uniprot (619 sequences), then concatenated into a single file of sequences (4817 sequences). The file was first filtered for sequences that share more than 95% similarity (2240 sequences). After filtering by similarity, the sequences were filtered for fragments and those of length less than 300 amino acids were removed (764 sequences). Finally, the canonical chorismate synthase (PDB identifier: 1R52 [28]) was added to yield a dataset of 765 sequences.

#### GPCR

Class-A rhodopsin-like G-protein coupled receptor sequences were downloaded from www.gpcrs.org (5025 sequences). The sequences were first filtered to remove sequences much longer than the average; those larger than 500 amino acids were removed (4786 sequences). Sequences more than 95% similar were then removed (2475 sequences). Finally, the canonical G-protein coupled receptor (PDB identifier: 2VT4 [29]) was added to yield a dataset of size 2476.

#### PDZ

Sequences of proteins containing PDZ domains were downloaded from the Uniprot headers indicated in Pfam entry PF00595 (4681 sequences) [16]. The PDZ domains were extracted, as indicated in PF00595, and those that were smaller than 65 residues or greater than 93 residues were removed (2561 sequences). Sequences more than 95% similar were then removed (1525 sequences). Finally, the canonical PDZ domain (PDB identifier: 1IU0 [30]) was added to yield a dataset of 1526 sequences.

### Coevolving residue algorithms

We treat the network of interactions among all paired positions as a weighted, undirected graph. The methods we use to obtain edge scores are described below; many of these methods have been previously published, and software to compute these scores is freely available. Hence, we refer the reader to the primary literature for the details of these methods.

#### Rand

Random is the simplest possible, and least likely to be successful, algorithm and is employed primarily as a baseline for both accuracy and reproducibility. In Rand, paired position scores are assigned random values drawn from a uniform distribution (*i.e.* every coupling lies uniformly in 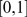).

#### OMES

Observed Minus Expected Squared is described in detail elsewhere [10], [15]. It essentially performs a chi-squared test on every possible pair of columns, looking for pairs of amino acids that occur more frequently that expected. “Expected” here means relative to the product of the frequencies of the amino acids in the individual columns of the alignment, which is equivalent to the assumption of no correlation between the two sites.

#### ELSC

Explicit Likelihood of Subset Co-variation is a perturbative algorithm that uses combinatorial arguments to explicitly calculate the probability that a random subset from a parent alignment has the observed amino acid profile at a given site. A thorough discussion of ELSC and its relation to oSCA, another perturbative algorithm, can be found elsewhere [12].

#### oSCA

Statistical Coupling Analysis (old) is a previously described method that looks for positions with changed residue compositions in sub-alignments relative to their parent alignment [11]. In this respect, it is a perturbative method in the style of ELSC [12]. These sub-alignments are made with respect to the most conserved residue in each column; hence the most conserved residue is calculated for each column, and the sub-alignment consists of all sequences with that conserved residue at that position. One way in which oSCA differs from all the other algorithms considered is that it generates a nonsymmetric score; 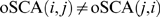. There are many possibilities in symmetrizing the oSCA score, and those methods could readily be compared via our pipeline sensitivity analysis. However, we will simply follow previous authors [15] and calculate only 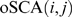 for 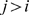.

#### nSCA

Statistical Coupling Analysis (new) is dramatically different from oSCA, so much so that they are more properly thought of as different algorithms [9]. The scoring method in nSCA is much closer to the relative column entropies, unlike oSCA, and is therefore symmetric.

#### MI

Edges in the MI graph have been assigned according to the mutual information between the two positions, defined for columns 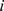 and 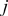 as
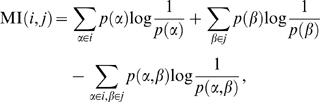
(3)where the sums are over the twenty possible residues at positions 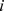 and 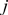. Hence, all that matters for calculating the MI between two positions are the individual and joint distributions of amino acids. The MI is a symmetric quantity. It was originally used for this purpose in [3], and many modifications of it have been proposed for coevolution and contact prediction [2], [3], [6]–[8].

#### MIp

Positional mutual information takes into account the background distributions of mutual information at two different positions by subtracting out a factor that is the product of the means of the two positional distributions, normalized by the average mutual information over the entire alignment [8].

#### ZNMI

In order to account for different alphabet sizes among columns in the multiple sequence alignment (*i.e.* columns that vary drastically by background entropy) we first normalize the MI (hereafter referred to as NMI) by the joint entropy (the third term of Eqn. 3), which reduces the correlation between MI and the product of the variances of the column MI [2]. To further correct for the differences in mean column NMI and variance of the column NMI, we make the assumption that the column NMI distribution can be approximated by a Gaussian distribution, 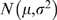, parameterized by the column NMI mean and variance; this approximation turns out to be reasonable when comparing it to a Gaussian distribution of equivalent size using a two-sample Kolmogorov-Smirnov test (CS, PDZ, and GPCR datasets, not shown). Given that the NMI distribution of column 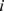 can be written as 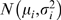 and the NMI distribution of column 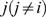 can be written as 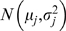, it is straightforward to show that
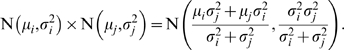
(4)This approximation has two main advantages. The first is that the closed-form solution makes the calculation easy to compute and computationally fast, as only the mean and variance of the column NMI must be calculated (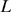 calculations where 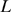 is the length of the pertinent columns in the multiple sequence alignment). The second advantage is that the calculation has a very intuitive interpretation. For columns 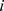 and 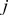, NMI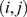 is considered significant if it is sufficiently large given that it comes from the column NMI of 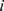 and column NMI of 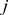. Values of NMI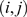 that are found between the column distributions of columns 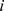 and 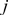 would be insignificant, whereas values of NMI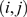 that are very far to the right of both column distributions would be considered significant. Finally, a z-score is calculated for the product NMI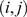 in Eqn. 4, leading us to the final metric referred to as ZNMI.

#### Zres

Z-scored residual mutual information first computes a linear regression of mutual information against the product of the means of the positional mutual information distributions. Afterwards, the residuals are z-scored against both residual positional distributions, and the product of those z-scores is computed (taking into account the sign of both z-scores) [6].

### Jaccard Indices

The Jaccard index is a classic, simple metric for comparing sets [47]. For two sets 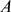 and 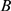, it is defined as the cardinality of the intersection divided by the cardinality of the union
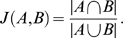
(5)The index is in 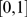; two sets of equal size sharing half their items have 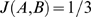, and two sets of equal size having a quarter of their items in common yield 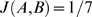. Our application of the Jaccard index to produce Figure 4 is as follows. We compute the consensus, weighted graphs for two scoring metrics. We prune the consensus graphs for different scoring methods at a given cutoff (see “Methods”) and ignore the weights of the remaining edges. The two sets in this case are then the set of edges for each graph, and the Jaccard index is readily computed. We repeat this calculation for multiple cutoffs (0.25, 0.5, 0.75) to obtain the heatmaps in Figure 4.

### Code Implementation

All of the algorithms, pipeline framework, and plotting were implemented in Python (www.python.org), with exception to OMES, McBASC, ELSC, and oSCA. Java code for these algorithms was downloaded from Anthony Fodor's homepage (www.afodor.net) and wrapped into our framework. All of the other algorithms were implemented as described in the relevant references. All of our code is available upon request, however we will not be responsible for the prerequisite Python and Python module implementations that our framework is dependent upon (*i.e.* NumPy, SciPy, networkx, etc.).

## Supporting Information

Figure S1 Comparison of MST and TNm1 graphs created from splits of the data. The MST and TNm1 graphs for a single split of the PDZ dataset (1526 sequences) are shown for contrast. The graph layouts in splits A and B are approximately the same so topological comparisons can be made by eye. Nodes that are in the intersection of all four graphs are colored green, while any node not in each and every graph is colored red. Similarly, edges that are common to all four graphs are drawn with thick lines. One can see that a common subgraph (green nodes connected by bold edges) is present, but consists of only a small fraction of the total number of nodes and edges. This illustrates the fact that MSTs and TNm1 graphs are by construction composed of very different residue-residue couplings.
(9.44 MB TIF)

Figure S2 Reproducibilty and accuracy for four MI-based algorithms on three different families. Scatterplots and histograms of reproducibility and accuracy for the three protein families (PDZ, 1256 sequences, CS, 765 sequences, GPCR, 2476 sequences) we consider in the text. The four MI-based algorithms compared are MI (green), MIp (red), ZNMI (blue), and Zres (black). The top row shows the results when we construct the consensus network using MST, and the bottom row with TNm1. The y axes on the reproducibility histograms have been rescaled to allow better visualization of the shapes of the distributions. While all three algorithms (MIp, ZNMI, and Zres) are improvements upon MI, MIp and ZNMI are comparable in their performance and Zres outperforms both ZNMI and MIp in two of three datasets.
(9.44 MB TIF)

Figure S3 Increasing accuracy without decreases in linear sequence separation. Shown here is the accuracy versus mean linear sequence separation for 150 splits for the full PDZ, CS, and GPCR datasets using MST as the pruning method (datasets are indicated in each plot with the number of sequences in parentheses). The color key shown in the lower right is used consistently throughout. While increasing the accuracy can reflect more pairs close in sequence, the strongest effect is in the PDZ dataset and is likely the effect of small sequence size. Note for CS and GPCR there can be dramatically different accuracies for roughly the same average sequence proximity.
(9.44 MB TIF)

Figure S4 Accuracy and reproducibility increase with increasing number of ‘informative’ sequences'. Scatterplots and histograms of reproducibility and accuracy for 150 spits of the PDZ, CS, and GPCR datasets with the ZNMI method (MSTs), shown as the number of sequences used in the alignments varies. Increasing the number of informative sequences — sequences that are dissimilar from the sequences that are already in your dataset — increases both the accuracy and reproducibility, though it is interesting to note that as more sequences are used the marginal gains in accuracy decrease faster than the marginal gains in reproducibility.
(9.44 MB TIF)

Figure S5 Changing the alignment method has minimal change on the resulting accuracy and reproducibility. Scatterplots and histograms of reproducibility and accuracy for 150 spits of the PDZ, CS, and GPCR datasets using MST as the pruning method (datasets are indicated in each plot with the number of sequence in parentheses) are shown for an initial alignment made with MUSCLE (top row) and MAFFT (bottom row). A quick comparison between the top row and bottom row shows that the changing between these two alignment methods has little affect on the accuracy and reproducibility for most of the algorithms.
(9.44 MB TIF)

Figure S6 Weak couplings are generally noisy and inaccurate. In this analysis we subjected the three full protein family datasets (PDZ, 1526 sequences, CS, 765 sequences, GPCR 2476 sequences) to our pipeline analysis, but in constructing the consensus network we have chosen the *smallest* N-1 edges, rather than using the MST or largest N-1 edges. oSCA has been omitted from the CS panel, as it could not be calculated due to numerical instability. For all algorithms and all three protein families, the accuracy suffers. In general, the reproducibility is also quite a bit lower. However, it is interesting to note that oSCA is *more* reproducible in this case, and OMES in the GPCR panel still has high reproducibility. The first observation highlights oSCA as an “outlier” in terms of scoring metric, and the second points strongly to the need to consider reproducibility and accuracy in tandem.
(9.44 MB TIF)
