# Supplementary figures and images for: Validation of Coevolving Residue Algorithms via Pipeline Sensitivity Analysis: ELSC and OMES and ZNMI, Oh My!
Source: PLoS One. 2010 Jun 1;5(6):e10779. doi: 10.1371/journal.pone.0010779 (PMC2879359; doi:10.1371/journal.pone.0010779)

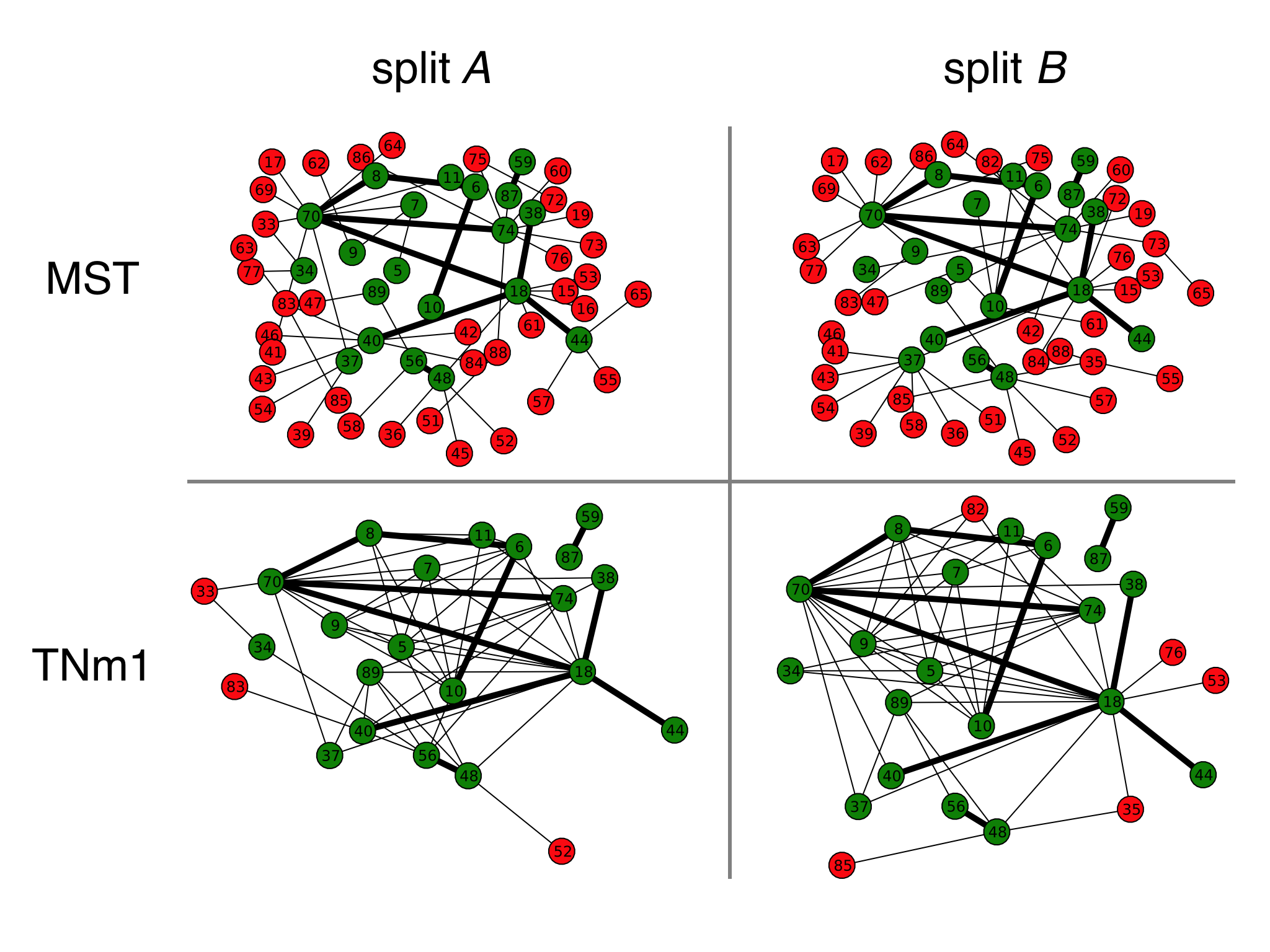

Supplement: Figure S1 — Comparison of MST and TNm1 graphs created from splits of the data. The MST and TNm1 graphs for a single split of the PDZ dataset (1526 sequences) are shown for contrast. The graph layouts in splits A and B are approximately the same so topological comparisons can be made by eye. Nodes that are in the intersection of all four graphs are colored green, while any node not in each and every graph is colored red. Similarly, edges that are common to all four graphs are drawn with thick lines. One can see that a common subgraph (green nodes connected by bold edges) is present, but consists of only a small fraction of the total number of nodes and edges. This illustrates the fact that MSTs and TNm1 graphs are by construction composed of very different residue-residue couplings. (9.44 MB TIF) [file pone.0010779.s001.tif]

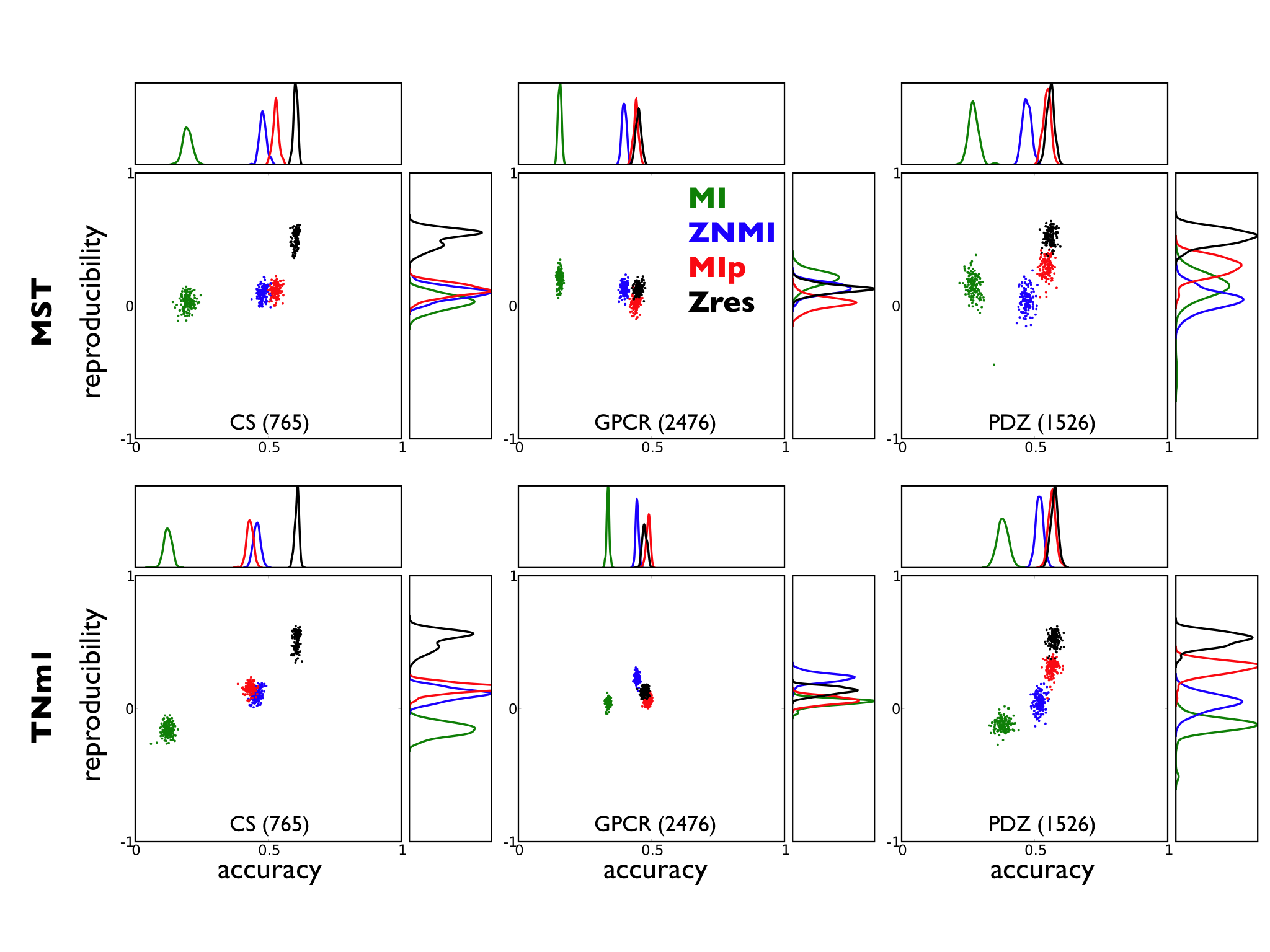

Supplement: Figure S2 — Reproducibilty and accuracy for four MI-based algorithms on three different families. Scatterplots and histograms of reproducibility and accuracy for the three protein families (PDZ, 1256 sequences, CS, 765 sequences, GPCR, 2476 sequences) we consider in the text. The four MI-based algorithms compared are MI (green), MIp (red), ZNMI (blue), and Zres (black). The top row shows the results when we construct the consensus network using MST, and the bottom row with TNm1. The y axes on the reproducibility histograms have been rescaled to allow better visualization of the shapes of the distributions. While all three algorithms (MIp, ZNMI, and Zres) are improvements upon MI, MIp and ZNMI are comparable in their performance and Zres outperforms both ZNMI and MIp in two of three datasets. (9.44 MB TIF) [file pone.0010779.s002.tif]

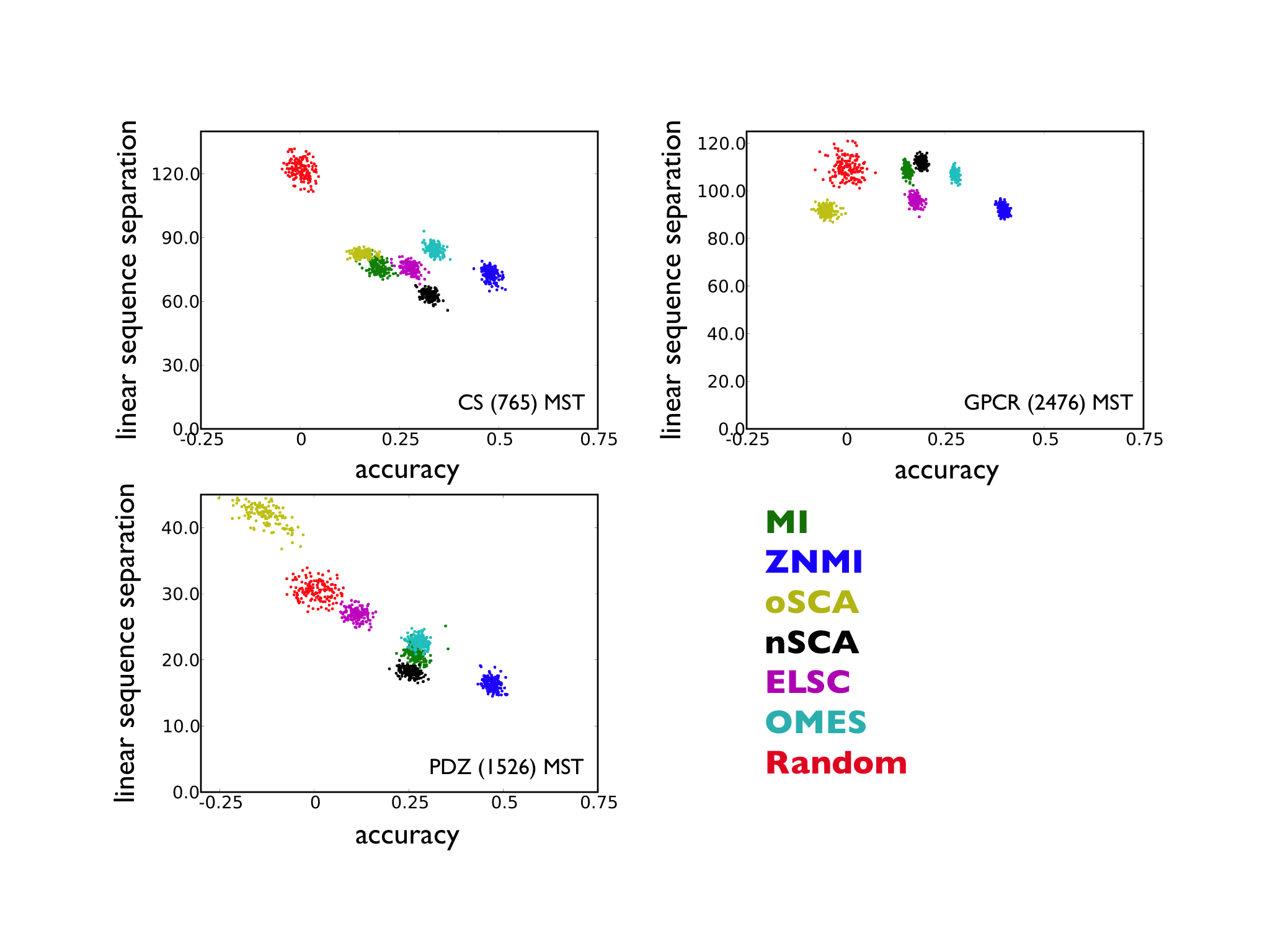

Supplement: Figure S3 — Increasing accuracy without decreases in linear sequence separation. Shown here is the accuracy versus mean linear sequence separation for 150 splits for the full PDZ, CS, and GPCR datasets using MST as the pruning method (datasets are indicated in each plot with the number of sequences in parentheses). The color key shown in the lower right is used consistently throughout. While increasing the accuracy can reflect more pairs close in sequence, the strongest effect is in the PDZ dataset and is likely the effect of small sequence size. Note for CS and GPCR there can be dramatically different accuracies for roughly the same average sequence proximity. (9.44 MB TIF) [file pone.0010779.s003.tif]

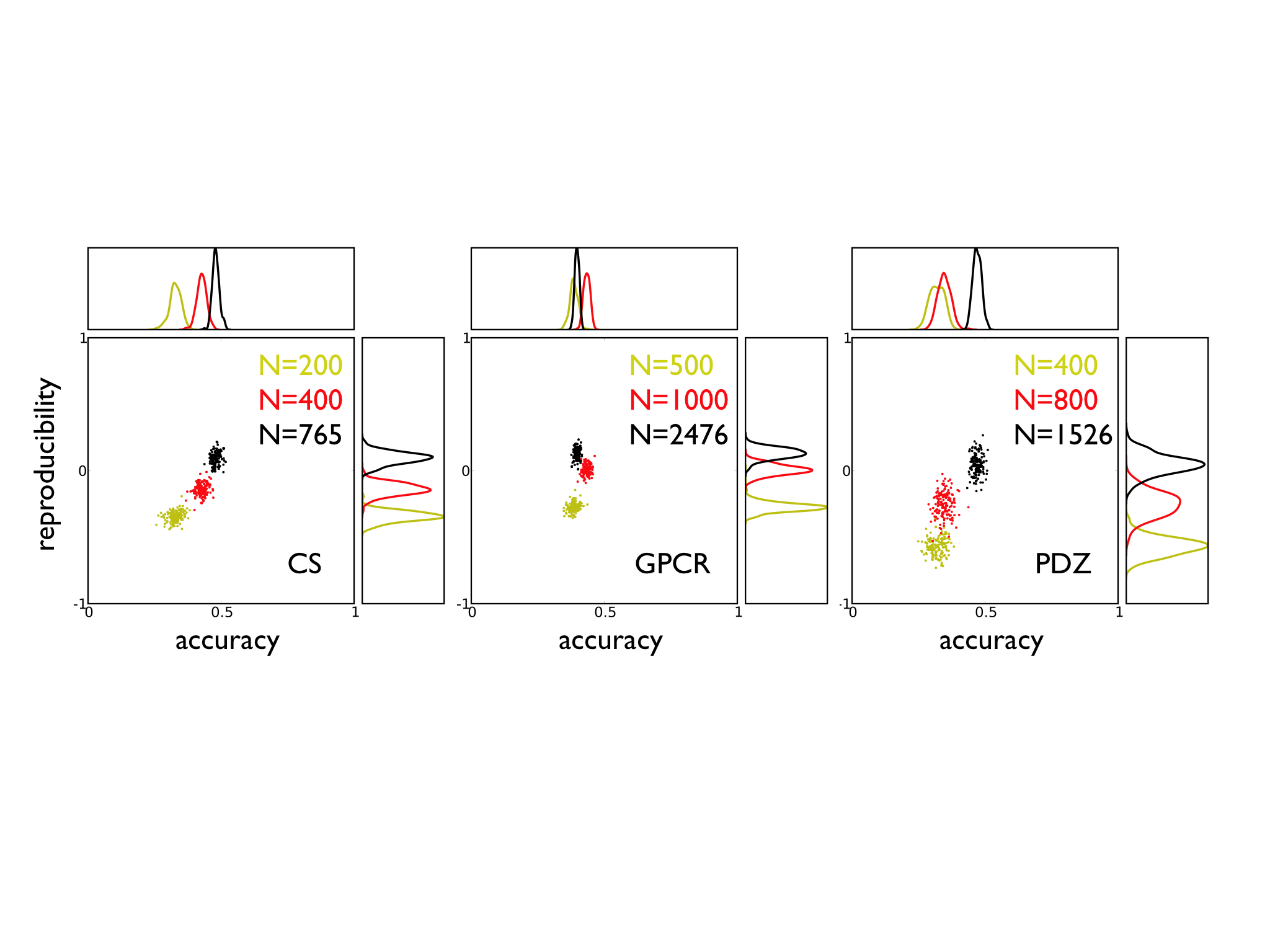

Supplement: Figure S4 — Accuracy and reproducibility increase with increasing number of ‘informative’ sequences'. Scatterplots and histograms of reproducibility and accuracy for 150 spits of the PDZ, CS, and GPCR datasets with the ZNMI method (MSTs), shown as the number of sequences used in the alignments varies. Increasing the number of informative sequences — sequences that are dissimilar from the sequences that are already in your dataset — increases both the accuracy and reproducibility, though it is interesting to note that as more sequences are used the marginal gains in accuracy decrease faster than the marginal gains in reproducibility. (9.44 MB TIF) [file pone.0010779.s004.tif]

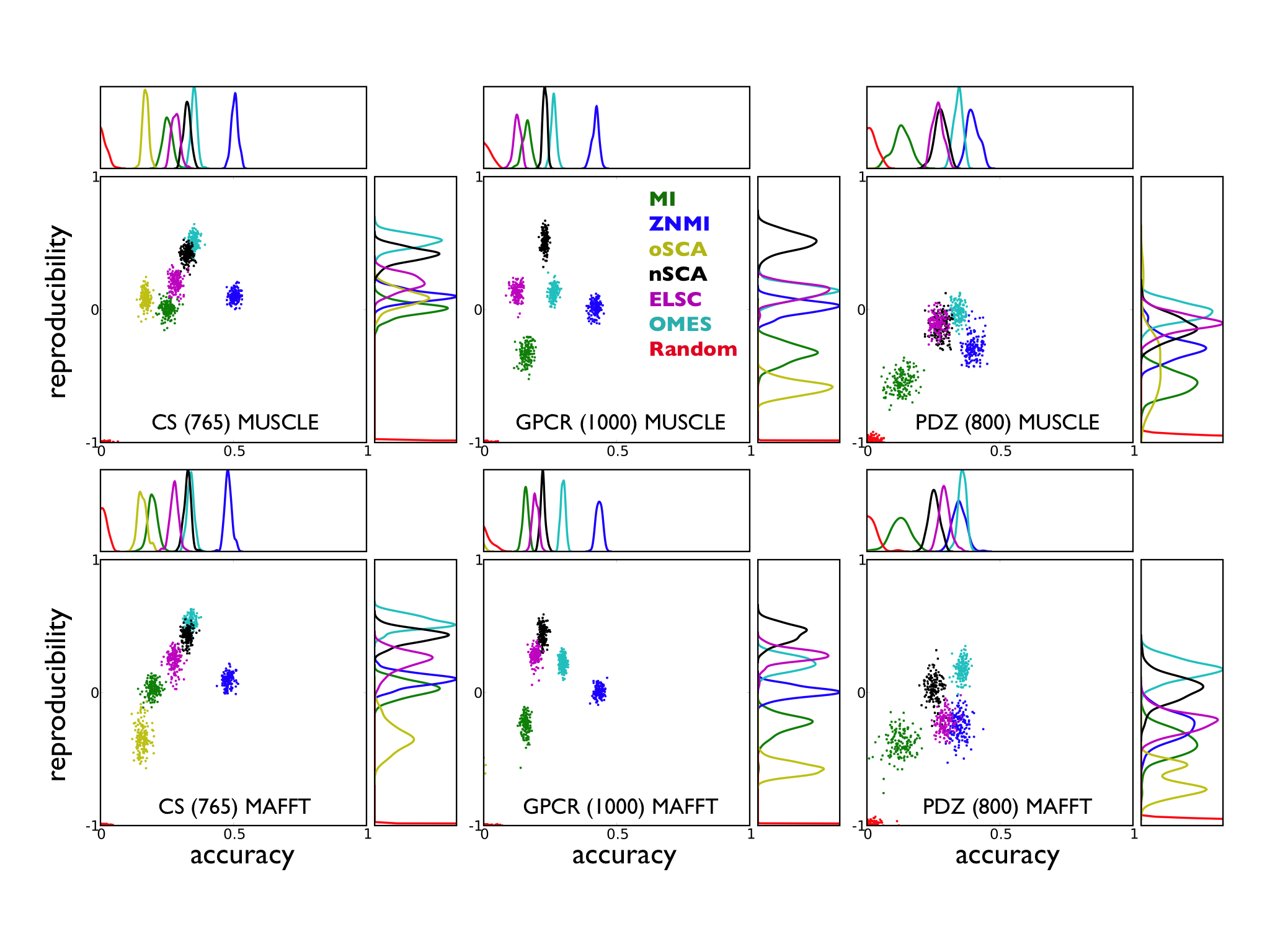

Supplement: Figure S5 — Changing the alignment method has minimal change on the resulting accuracy and reproducibility. Scatterplots and histograms of reproducibility and accuracy for 150 spits of the PDZ, CS, and GPCR datasets using MST as the pruning method (datasets are indicated in each plot with the number of sequence in parentheses) are shown for an initial alignment made with MUSCLE (top row) and MAFFT (bottom row). A quick comparison between the top row and bottom row shows that the changing between these two alignment methods has little affect on the accuracy and reproducibility for most of the algorithms. (9.44 MB TIF) [file pone.0010779.s005.tif]

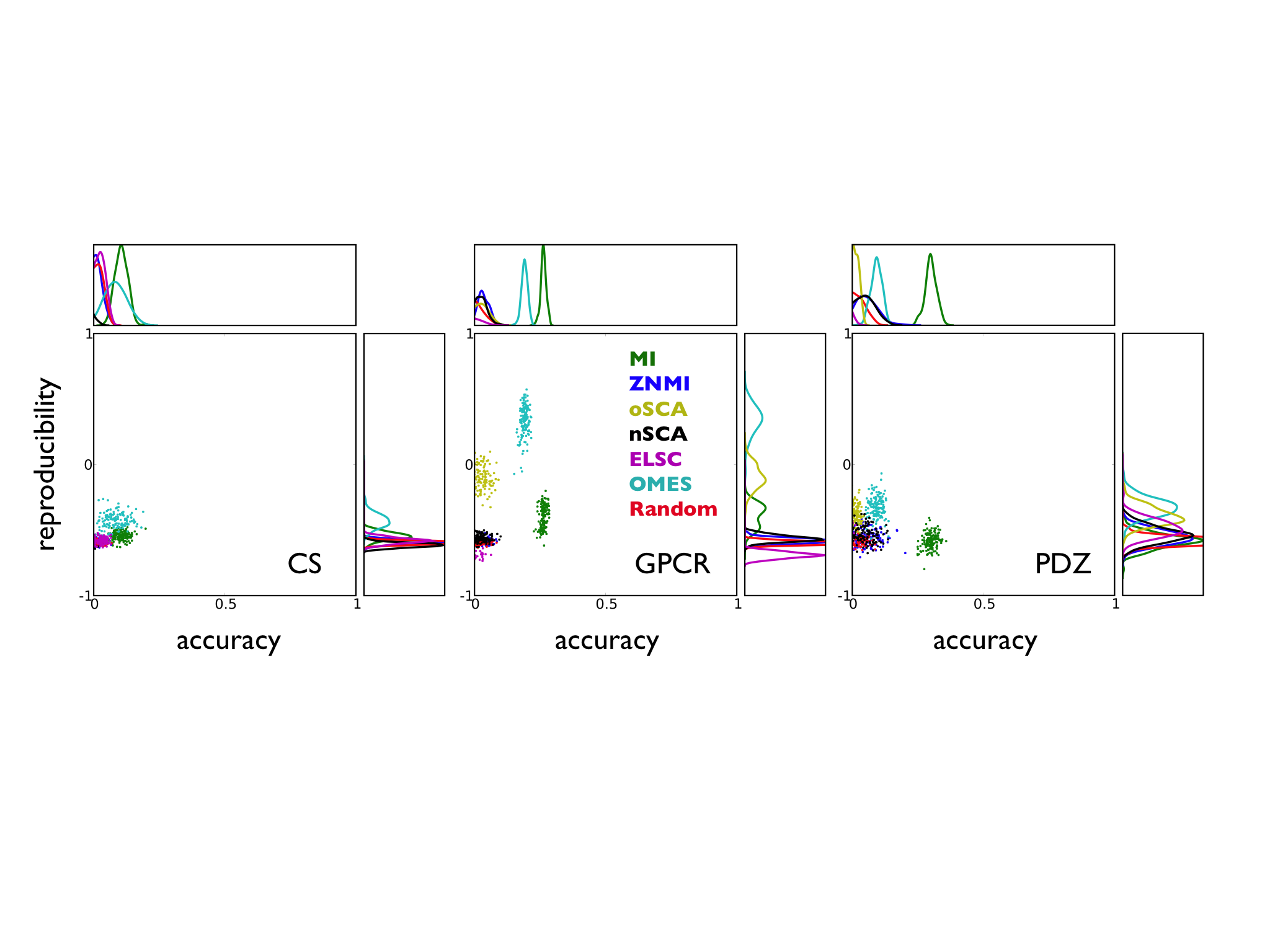

Supplement: Figure S6 — Weak couplings are generally noisy and inaccurate. In this analysis we subjected the three full protein family datasets (PDZ, 1526 sequences, CS, 765 sequences, GPCR 2476 sequences) to our pipeline analysis, but in constructing the consensus network we have chosen the smallest N-1 edges, rather than using the MST or largest N-1 edges. oSCA has been omitted from the CS panel, as it could not be calculated due to numerical instability. For all algorithms and all three protein families, the accuracy suffers. In general, the reproducibility is also quite a bit lower. However, it is interesting to note that oSCA is more reproducible in this case, and OMES in the GPCR panel still has high reproducibility. The first observation highlights oSCA as an “outlier” in terms of scoring metric, and the second points strongly to the need to consider reproducibility and accuracy in tandem. (9.44 MB TIF) [file pone.0010779.s006.tif]
